# Phlorotannins from Phaeophyceae: Structural Diversity, Multi-Target Bioactivity, Pharmacokinetic Barriers, and Nanodelivery System Innovation

**DOI:** 10.3390/molecules30244733

**Published:** 2025-12-11

**Authors:** Joanna Harasym, Patryk Słota, Ewa Pejcz

**Affiliations:** 1 Adaptive Food Systems Accelerator-Science Centre, Wroclaw University of Economics and Business, Komandorska 118/120, 53-345 Wroclaw, Poland; joanna.harasym@ue.wroc.pl; 2 Department of Biotechnology and Food Analysis, Wroclaw University of Economics and Business, Komandorska 118/120, 53-345 Wroclaw, Poland; patryk.slota@ue.wroc.pl

**Keywords:** phlorotannins, brown macroalgae, bioavailability, nanodelivery systems, encapsulation, antioxidant activity, marine biotechnology, functional ingredients

## Abstract

Phlorotannins, a unique group of polyphenolic compounds derived exclusively from brown macroalgae (Phaeophyceae), have gained substantial scientific and industrial interest due to their structural diversity and multifaceted bioactivities. These marine metabolites, composed of phloroglucinol units linked through various C–C and C–O–C bonds, exhibit broad-spectrum antioxidant, anti-inflammatory, antimicrobial, antidiabetic, anticancer, and neuroprotective effects. Despite their promising in vitro efficacy, large-scale application remains hindered by three critical translational barriers: (i) extreme natural variability in phlorotannin content driven by ecological and seasonal factors, complicating raw material standardization; (ii) physicochemical instability and poor aqueous solubility resulting in limited oral bioavailability; and (iii) insufficient development of advanced delivery systems to ensure controlled release and targeted bioactivity. This comprehensive review integrates ecological, biochemical, and technological perspectives to establish a unified framework for translating phlorotannin research toward clinical and commercial realization. It systematically examines biosynthetic regulation, structural classification, extraction and purification methods, bioactivity mechanisms, pharmacokinetic barriers, and toxicological safety considerations. The review concludes by highlighting future research priorities essential for achieving industrial scalability, formulation reproducibility, and regulatory acceptance in marine bioactive development.

## 1. Introduction

Phlorotannins are phenolic secondary metabolites derived almost exclusively from brown macroalgae (Phaeophyceae), distinguishing them from terrestrial plant polyphenols [1,2,3]. These compounds are dehydro-polymers of phloroglucinol (1,3,5-trihydroxybenzene), differing substantially from terrestrial tannins [4,5]. Their specific structure reflects biosynthetic pathways, ecological functions, and broad biological activities [6,7,8]. Primary sources belong to Fucaceae, Sargassaceae, and Alariaceae families, with *Ecklonia cava*, *Fucus vesiculosus*, and *Ascophyllum nodosum* extensively studied [1,9,10]. These compounds serve as chemical defence mechanisms against herbivory, oxidative stress from UV radiation, desiccation, and microbial colonisation [11,12,13]. Phlorotannins represent valuable bioactive compounds with therapeutic potential for food, pharmaceutical, cosmetic, and nutraceutical applications [6,14,15,16].

Scientific exploration began in the 1970s when phenolic metabolites were first identified in brown algae [7]. Originally termed phaeophyte tannins or polyphloroglucinols, these metabolites represent the youngest identified class of plant polyphenolics [7,14]. Advances in HPLC-MS, NMR spectroscopy, and fractionation techniques have progressively elucidated their molecular architecture [4,16,17]. Early findings documented antioxidant, anti-inflammatory, and antimicrobial activities, stimulating interest in pharmacological applications [13,14,18]. Recent reviews have systematically documented extraction methodologies, structural classification, and broad-spectrum bioactivities [1,2,14,19].

While progress has been made in characterising chemical structures and documenting in vitro biological activities, critical knowledge gaps limit translation to clinical and commercial applications [6,14]. Three interconnected challenges remain to successfully proceed with the implementation:

- The bioavailability problem, wherein phlorotannins exhibit poor oral bioavailability due to limited solubility, chemical instability, and extensive first-pass metabolism [6,20,21];
- Raw material standardization issues, with natural variability of 0.5–30% of dry weight complicating industrial quality control [11,19];
- The delivery system bottleneck, where advanced formulation strategies remain at proof-of-concept stages [6,21].

This review aims to establish a translational framework bridging documented in vitro activities and clinical realization by addressing the bioavailability paradox through advanced delivery system innovation. Specific objectives include:

(1) Elucidating ecological origins and biosynthetic regulation affecting raw material standardization;
(2) Characterizing chemical architecture and structure–activity relationships;
(3) Reviewing extraction, purification, and analytical methodologies emphasizing green technologies;
(4) Analyzing multi-target bioactive mechanisms including antioxidant, anti-inflammatory, antidiabetic, anticancer, neuroprotective, and antimicrobial activities;
(5) Examining the bioavailability paradox including physicochemical properties, gastrointestinal modifications, and safety considerations;
(6) Evaluating advanced delivery systems including polymeric nanoparticles, liposomal systems, electrospun nanofibers, and pH-responsive platforms;
(7) Addressing industrial translation challenges including controlled aquaculture, heavy metal contamination, purification economics, and regulatory pathways.

## 2. Ecological Role and Chemical Structure of Phlorotannins

### 2.1. Ecological Role and Biosynthetic Regulation

Phlorotannins mitigate oxidative stress in intertidal macroalgae exposed to intense sunlight, high temperatures, and desiccation, preventing tissue damage and excessive reactive oxygen species (ROS) production [1,11]. These compounds function as electron traps due to numerous hydroxyl groups in their polymeric structure [1,3]. The high oligomerisation degree represents an evolutionary adaptation optimising antioxidant defence efficiency, underlying the potent in vitro antioxidant and cytoprotective activities observed in mammalian studies [1,2].

Phlorotannin biosynthesis responds dynamically to ecological conditions [11,19]. Abiotic factors—water temperature, light intensity, salinity, nutrient availability, and tidal patterns—alongside biotic pressures including herbivory, epiphyte colonization, and reproductive status, influence phenolic production rates and structural profiles [11,12,19]. This environmental sensitivity creates natural variability in phlorotannin content (0.5–30% dry weight), complicating industrial standardization [11,19,22]. This necessitates advanced chemical fingerprinting or controlled aquaculture cultivation, increasing production complexity and costs [19,22].

Phlorotannin biosynthesis is governed by type III polyketide synthase (PKS1), which catalyzes phloroglucinol monomer formation from malonyl-CoA through sequential condensation reactions [6,23]. Monomers subsequently polymerise through various linkages to form structurally diverse phlorotannins [23]. Pathways have been elucidated in *Ectocarpus siliculosus* as a model [23]. Meslet-Cladière et al. (2013) characterized PKS1 through crystallographic analysis at 2.85 Å resolution, revealing a modified cysteine residue covalently linked to a long-chain acyl group and an additional substrate-binding pocket absent in other type III polyketide synthases [23]. PKS1 gene expression positively correlated with phlorotannin accumulation in *E. siliculosus* strains transitioning from freshwater to seawater, indicating transcriptional regulation during environmental adaptation [23]. Phylogenetic analyses suggest PKS1 emerged via lateral gene transfer from actinobacteria [23]. This molecular understanding enables metabolic engineering strategies for enhanced yield and targeted structural profiles [6,23].

**Figure 1:**
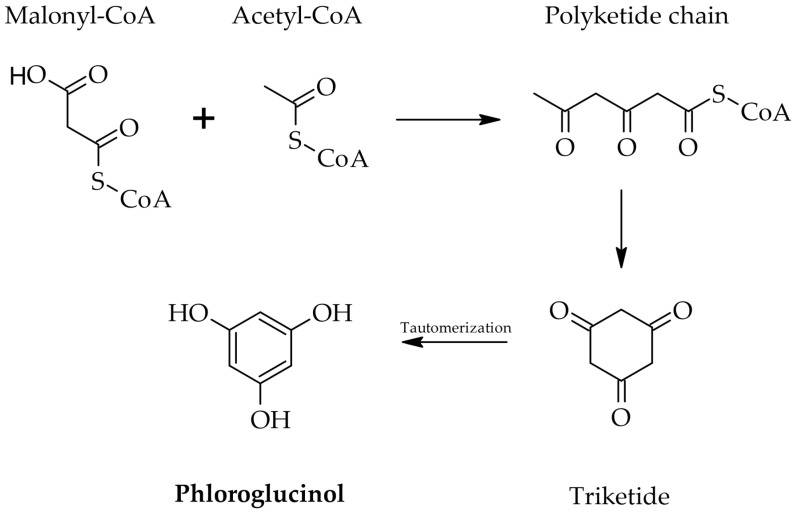
Proposed biosynthetic pathway of phlorotannins in brown algae, showing the involvement of the acetate–malonate pathway and the role of type III polyketide synthase enzymes (PKS1) [24,25].

As illustrated in Figure 1, biosynthesis proceeds through PKS1-catalyzed condensation of malonyl-CoA units, followed by tautomerization to yield phloroglucinol monomers, which undergo oxidative polymerization to generate diverse oligomeric structures [23,24,25].

### 2.2. Structural Diversity and Classification

**Figure 2:**
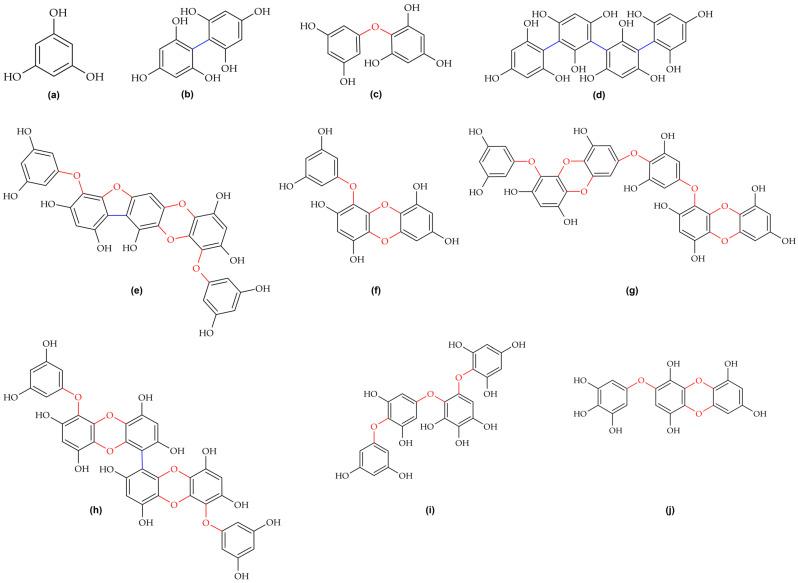
Representative chemical structures of main phlorotannin classes: (**a**) phloroglucinol, (**b**) fucol, (**c**) diphloroethol, (**d**) tetrafucol-A, (**e**) phlorofucofuroeckol-B, (**f**) eckol, (**g**) dieckol, (**h**) 6,6′-bieckol, (**i**) fuhalol, and (**j**) carmalol [23,24,25,26]. Red lines depict oxygen bridges; blue lines depict carbon direct linkages.

Phlorotannins are dehydro-polymers of phloroglucinol (1,3,5-trihydroxybenzene) [4]. The phloroglucinol core yields polymerisation patterns that distinguish phlorotannins from plant polyphenols based on gallic or ellagic acid [4,5]. Molecular weights range from 126 Da to over 100,000 Da, with commercial extracts typically spanning 10,000–100,000 Da [4,19]. Structural complexity arises from diverse covalent linkages between phloroglucinol units through carbon–carbon (C–C) and carbon–oxygen–carbon (C–O–C) bonds [2,4]. Six major subclasses are defined based on linkage polymorphism, determining chemical reactivity, physicochemical properties, and biological activities [2,18]. Fucols contain C–C phenyl bonds creating biphenyl or triphenyl structures [2,4]. Phlorethols and fuhalols feature C–O–C ether bonds with varying para- and ortho-linkages [2,4]. Fucophlorethols combine both linkage types, exemplified by phlorofucofuroeckol-B [1,2]. Eckols and carmalols contain fused dibenzo-1,4-dioxin ring systems conferring rigidity and enabling molecular target recognition, exemplified by dieckol, eckol, and 6,6′-bieckol [2,23,24,25,26]. These structures (Figure 2) enable precise molecular binding for signalling pathway and transcription factor inhibition [1,6].

Biological activity depends on polymerisation degree and molecular weight [1,19]. Lower-molecular-weight oligomers exhibit better membrane permeability and cellular uptake, whereas higher-molecular-weight polymers demonstrate superior radical-scavenging properties due to the increased number of hydroxyl groups [1]. Antioxidant potency generally increases with polymerisation degree up to an optimal range [1].

The structural characteristics present analytical challenges [4,19]. NMR spectroscopy, including HSQC and HMBC techniques, maps carbon–hydrogen correlations and identifies specific classes within complex mixtures [4]. LC-MS profiling has identified over 30 phlorotannin series within a single algal sample, revealing post-synthetic modifications, including acetylation, hydroxylation, and oxidation [27,28]. Despite approximately 150 distinct phlorotannins being isolated over the past four decades, standardised quantification methods remain scarce [7,18]. Structural complexity directly dictates the selection of extraction methodology and the optimisation of purification strategy [7,16].

## 3. Extraction, Purification, and Analytical Characterization

Efficient phlorotannin extraction from brown seaweeds is fundamental for pharmaceutical, nutraceutical, and cosmetic applications [16,21]. Traditional methods rely on solvent-based extraction using acetone, ethyl acetate, and methanol in aqueous mixtures [16,29]. Extraction efficiency depends on the solvent polarity, seaweed species, harvest season, and drying conditions [10,16]. Sustainability considerations drive development of greener extraction alternatives reducing solvent consumption, energy use, and processing time [10,30].

Ultrasound-assisted extraction (UAE) and microwave-assisted extraction (MAE) increase phlorotannin yield, reduce extraction time, and minimise solvent consumption [10,30]. These approaches enhance cell wall disruption while preserving chemical stability and bioactivity [21]. Amarante et al. (2020) demonstrated that optimised MAE conditions—57% (*v*/*v*) ethanol, 75 °C, 5 min—achieved phlorotannin recovery comparable to conventional hydroacetonic extraction [30].

Hydroethanolic MAE extract showed higher phlorotannin concentration (11.1 ± 1.3 mg phloroglucinol equivalents (PGE)/g dry weight (DW)) versus conventional extract (9.8 ± 1.8 mg PGE/g DW), with similar constituents including tetrafucol, pentafucol, hexafucol, and heptafucol derivatives [30]. UAE utilizes acoustic cavitation to disrupt algal cell walls, offering reduced extraction time and decreased solvent consumption [16]. Gisbert et al. (2023) demonstrated that parameter optimisation is essential for maximising yields from *A. nodosum* [10]. Supercritical fluid extraction (SFE) using carbon dioxide offers non-toxicity, environmental compatibility, and selective extraction capabilities [30]. Optimizing MAE, UAE, and SFE is critical for commercializing phlorotannin ingredients at scale [10,21,30].

Seasonality and species variability lead to significant fluctuations in phlorotannin content [16,31]. Ford et al. (2020) demonstrated substantial temporal variation using Folin–Ciocalteu assays with quantitative NMR spectroscopy [31]. Complex macroalgal matrices rich in polysaccharides render the isolation of phenolics challenging, with structural diversity further complicating the extraction process [7,16]. Santos et al. (2019) comprehensively reviewed extraction methodologies, underscoring the importance of method selection for preserving bioactivity [16].

Crude extracts contain heterogeneous compound mixtures, necessitating purification and fractionation [16,29]. Separation strategies utilize polarity and molecular size as primary parameters [29]. Initial techniques include liquid–liquid partitioning, dialysis, and ultrafiltration [16,29]. Chen et al. (2023) employed ethyl acetate partitioning and UPLC-MS/MS to identify fifty-nine chromatographic peaks, of which fifteen were structurally elucidated as phlorotannin isomers or derivatives [32]. Biological activity depends on polymerization degree, with lower-molecular-weight oligomers exhibiting better membrane permeability and higher-molecular-weight polymers possessing superior radical-scavenging properties [1,19,29]. Preparative and analytical HPLC enables molecular separation based on size, polarity, and affinity [7,18]. Isaza Martínez and Torres (2013) reviewed four decades of evolution in chromatographic techniques [7]. High-speed counter-current chromatography (HSCCC) provides efficient purification without irreversible adsorption [33]. Zhou et al. (2019) combined HSCCC and Sephadex LH-20 to isolate neuroprotective eckmaxol from *Ecklonia maxima* [33]. Activity-guided fractionation couples chromatographic separation with biological screening [34,35]. Sugiura et al. (2006) isolated anti-allergic phlorofucofuroeckol-B from *Eisenia arborea* [34], while Cho et al. (2019) isolated antiviral fractions from E. cava using H1N1 influenza screening [35].

The coelution of phlorotannin isomers remains a significant analytical challenge, affecting resolution and quantification accuracy [7,18]. Robust quantification methods and standardized quality control protocols remain scarce [7,18]. Table 1 summarizes main phlorotannin subclasses, algal sources, extraction techniques, and reported bioactivities.

### Structural Identification and Quantification

Precise identification and quantification of phlorotannins necessitate dedicated spectroscopic and analytical techniques to resolve structural complexity [4,18]. Analytical assessment is mandatory at every stage of extraction and purification to monitor process efficiency and ensure product quality [16,29]. Gisbert et al. (2023) emphasized integrating spectrophotometric and chromatographic techniques for comprehensive characterization [10].

Total phenolic content quantification commonly employs colorimetric assays including Folin–Ciocalteu (FC) and 2,4-dimethoxybenzaldehyde (DMBA) methods [18,29]. The FC assay relies on electron transfer reactions where phenolic hydroxyl groups reduce phosphotungstate-phosphomolybdate complexes, producing a spectrophotometrically measured blue chromophore [29]. However, these methods suffer from limited specificity and potential interference from non-phenolic substances [4,18]. Radical-scavenging potential is characterised using the DPPH assay [29,40]. Ford et al. (2020) advocated for mass spectrometric profiling for accurate characterisation [31].

NMR spectroscopy provides detailed insights into molecular framework, substitution patterns, and linkage types [4,40]. One-dimensional (^1^H NMR, ^13^C NMR) and two-dimensional techniques (HSQC, HMBC) map carbon–hydrogen correlations and identify structural classes, enabling determination of inter-unit linkage nature (C–C versus C–O–C bonds) [4]. Yotsu-Yamashita et al. (2013) employed high-resolution MS/MS and NMR analysis for structural determination of novel phlorotannins (974-A and 974-B) from *Ecklonia kurome* [40]. Ford et al. (2020) demonstrated the quantitative utility of NMR for determining phenolic content with compound-class selectivity and reduced matrix interference [31].

Mass spectrometry, particularly HPLC-MS/MS and UPLC-QE-MS/MS, enables the determination of molecular weight, analysis of fragmentation patterns, and structural assignments [32,35]. Cho et al. (2019) utilized HPLC-qTOF-MS with relative molecular descriptor assessments to characterize compounds from *E. cava*, with the EC70 fraction encompassing thirteen predicted phlorotannins, ten successfully identified [35]. Birkemeyer et al. (2020) identified over 30 phlorotannin series within a single algal sample, revealing post-synthetic modifications, including acetylation, hydroxylation, and oxidation [28]. Chen et al. (2023) identified fifty-nine chromatographic peaks using UPLC-QE-MS/MS, with fifteen structurally elucidated as phlorotannin isomers including eckol, fucofurodiphlorethol, and fucotriophlorethol [32].

FTIR identifies characteristic absorption bands: phenolic hydroxyl groups (3200–3600 cm^−1^), aromatic C=C stretches (1400–1600 cm^−1^), and C–O stretches (1000–1300 cm^−1^) [16]. UV-Vis spectroscopy detects absorption maxima (200–300 nm) from π→π* transitions in aromatic rings [10].

Quality control remains challenging due to natural variability from species differences, environmental factors, and extraction methodology [18,31]. Coelution of phlorotannin isomers results from three phenomena: positional isomerism, which creates compounds with nearly identical retention characteristics; oligomers of identical molecular weight but different linkage types, demonstrating overlapping hydrophobicity; and molecular weight distributions spanning 126 Da to over 100,000 Da, creating retention time continuums [4,7,19,28,32]. Ford et al. (2020) demonstrated quantitative results varying up to 3-fold depending on calibration standard selection [31]. An international consensus on reference standards and validated protocols remains urgently needed for regulatory acceptance [16,18,31].

## 4. The Bioavailability Problem and Pharmacokinetic Barriers

Despite extensive in vitro bioactivity documentation, phlorotannins face a critical translational bottleneck: the bioavailability paradox. This manifests as a discrepancy between pharmacological efficacy in experimental systems and limited therapeutic effectiveness following oral administration, attributable to physicochemical barriers, gastrointestinal degradation, metabolic transformations, and matrix-dependent absorption limitations [6,21,22].

Phlorotannin therapeutic effectiveness is determined not by inherent biological potency—demonstrated across antioxidant, anti-inflammatory, antidiabetic, anticancer, and neuroprotective mechanisms—but by successful delivery to target tissues at therapeutically relevant concentrations [1,22]. The primary constraint is exceptionally low bioavailability from synergistic physicochemical and metabolic hurdles [1,22]. Phlorotannins exhibit limited aqueous solubility and poor membrane permeability, restricting passive diffusion across gastrointestinal epithelial barriers [22]. They demonstrate extreme chemical lability, being susceptible to degradation during storage, manufacturing, and gastrointestinal transit due to exposure to light, oxidative conditions, elevated temperatures, and pH extremes [1,22]. Surviving molecules undergo extensive biotransformation by gut microbiota, which enzymatically cleave oligomeric structures, frequently rendering compounds pharmacologically inactive [22]. This necessitates strategic reorientation from bioactivity screening toward development of advanced delivery systems [6,22].

Numerous hydroxyl groups, critical for radical-scavenging activity, simultaneously render phlorotannins highly redox-active and prone to autoxidation, resulting in the formation of quinone intermediates and polymeric oxidation products [1,22]. Molecular weight distribution complicates absorption dynamics, with structure-permeability studies demonstrating inverse correlation between oligomer size and intestinal permeability [41]. Smaller oligomers exhibit superior absorption compared to larger polymeric fractions, which have restricted transcellular or paracellular transport capacity [41]. Bai et al. (2020) demonstrated that polyvinylpyrrolidone (PVP) nanoparticle encapsulation substantially improved oxidative stability, reduced environmental degradation, enhanced controlled-release kinetics, and preserved antioxidant activity [42].

Phlorotannin metabolic fate and systemic absorption kinetics remain under-characterized due to complex polymeric structures complicating analytical tracking [14,41]. Upon oral ingestion, phlorotannins undergo sequential biochemical transformations mediated by digestive enzymes and gut microbiota through hydrolytic cleavage, reductive metabolism, and conjugation reactions [41,43]. Corona et al. (2016) demonstrated that following colonic fermentation of *Ascophyllum nodosum*-derived phlorotannins, lower-molecular-weight oligomers are metabolised into absorbable phenolic derivatives with inter-individual variations reflecting gut microbiome differences [41]. Phase II metabolites, including glucuronide and sulfate conjugates, are detectable in plasma and urine following oral ingestion, confirming extensive conjugation in liver and intestine [41,44]. Pharmacokinetic parameters including Cmax, Tmax, and t1/2 have been quantitatively determined [44]. Sallam et al. (2021) elucidated how specific microbial metabolites—rather than intact parent structures—frequently serve as primary pharmacologically active entities [43].

Phlorotannin bioavailability is substantially influenced by binding interactions with dietary macromolecules and food matrix components [22]. Phlorotannins exhibit strong affinity for proteins, carbohydrates, and lipids through hydrogen bonding, hydrophobic interactions, and electrostatic associations, forming insoluble complexes that reduce bioaccessibility [22]. Arazo-Rusindo et al. (2025) demonstrated that matrix composition profoundly influences phlorotannin release, stability, and absorption potential [20]. Encapsulation strategies that isolate phlorotannins from reactive dietary components represent essential approaches for achieving consistent systemic exposure [6,21].

Phlorotannin-rich extract safety profiles are generally favorable [14,45]. Acute toxicity data provide quantitative safety margins: phlorotannin extracts demonstrated acute oral LD50 values exceeding 2000 mg/kg body weight in Sprague–Dawley rats, with acute dermal toxicity LD50 values greater than 2000 mg/kg [45]. In vitro cytotoxicity studies using human keratinocyte cell lines demonstrated no toxicity at concentrations up to 100 μg/mL [46]. The Cosmetic Ingredient Review Expert Panel (2022) concluded that brown algae-derived ingredients are safe for use in both leave-on and rinse-off products [45]. Shin et al. (2024) conducted pharmacokinetic investigations of *Ecklonia cava* phlorotannins following both intravenous and oral administration, revealing moderate oral bioavailability and the identification of specific metabolites [44].

However, comprehensive long-term toxicological evaluations remain incomplete, with gaps in chronic toxicity assessments, reproductive toxicity studies, genotoxicity profiling, and drug-nutrient interactions [14]. Okeke et al. (2021) emphasized the necessity for systematic toxicological investigations before clinical adoption for pharmaceutical indications [14]. Current safety knowledge supports the use of low-risk applications in food supplements and cosmetics; however, data deficiencies preclude advancement into pharmaceutical contexts, which require rigorous regulatory scrutiny [14,45].

## 5. Advanced Delivery Systems and Formulation Innovations

Advanced delivery systems represent critical enabling technologies for overcoming pharmacokinetic barriers and achieving therapeutic efficacy. Protective encapsulation platforms, controlled-release matrices, and targeted delivery vehicles address physicochemical instability, poor membrane permeability, gastrointestinal degradation, and rapid metabolic clearance [6,21,22].

### 5.1. Rationale and Design Principles

Encapsulation techniques address three objectives: protection of labile phlorotannin molecules from degradation mediated by light exposure, atmospheric oxygen, temperature fluctuations, and gastrointestinal conditions; control of release kinetics ensuring sustained therapeutic concentrations; and modulation of bioaccessibility through enhanced solubility and membrane permeability [22]. Particle size optimisation is critical, as nanoscale dimensions (50–200 nm) enhance cellular uptake through endocytic pathways and facilitate lymphatic absorption, bypassing first-pass metabolism [21,22]. Surface functionalization strategies, including PEGylation and ligand conjugation, enhance the precision of delivery [6]. Release kinetics engineering, achieved through polymer composition and environmental responsiveness (pH, temperature, and enzymatic activity), enables programmable delivery profiles [21,22].

### 5.2. Polymeric Nanoparticle Systems

Polymeric nanoparticles represent the most extensively investigated encapsulation platform, with carriers typically ranging from 50–500 nm fabricated from biocompatible polymers including poly(lactic-co-glycolic acid) (PLGA), polycaprolactone (PCL), polyvinylpyrrolidone (PVP), chitosan, and alginate [6,22]. Bai et al. (2020) demonstrated that PVP-encapsulated phlorotannin nanoparticles exhibited enhanced oxidative stability, sustained controlled-release kinetics, preserved antioxidant activity, and absence of cytotoxicity toward human keratinocyte cell lines [42]. Tong et al. (2021) reviewed nano-delivery platforms for diabetes applications, demonstrating enhanced capacity to modulate α-glucosidase, α-amylase, and pancreatic lipase through improved cellular bioavailability [21]. Significant challenges persist: manufacturing costs for GMP-grade PLGA nanoparticles exceed $500–1000 per gram, and no phlorotannin-nanoparticle formulation has progressed beyond Phase I trials [6,21].

### 5.3. Liposomal and Vesicular Delivery Systems

Liposomal systems, comprising phospholipid bilayers, offer biocompatibility, the capacity to encapsulate both hydrophilic and lipophilic compounds, protection from enzymatic degradation, and clinically established safety profiles [6]. PEGylation generates sterically stabilised liposomes with extended circulation half-lives [6]. Nanogels combine nanoscale dimensions with high water content and swelling-responsive behavior [6]. Duan et al. (2023) demonstrated that liposomal, nanoparticle, and nanogel systems collectively address bioavailability challenges while enabling targeted delivery and controlled release [6].

### 5.4. Electrospun Nanofibers

Electrospun nanofiber scaffolds enable localised, sustained delivery for wound healing and tissue engineering applications [6]. Phlorotannin incorporation can be achieved through direct blending, coaxial electrospinning, surface immobilisation, or emulsion electrospinning [6]. Applications include wound dressings providing sustained antioxidant and antimicrobial activity, tissue engineering scaffolds, transdermal patches, and implantable devices [6].

### 5.5. pH-Responsive and Targeted Release Systems

pH-responsive systems exploit gastrointestinal pH gradients varying from gastric conditions (pH 1–3) through proximal small intestine (pH 6–7) to distal ileum and colon (pH 7–8) [6]. Pathological tissues exhibit acidic microenvironments (pH 6.0–6.8) compared to normal physiological pH (7.4) [6]. Enteric coating polymers, including the Eudragit series, protect acid-labile phlorotannins while enabling their release in the intestine [6,21]. Colonic delivery systems targeting pH values exceeding 7.0 hold promise for the treatment of inflammatory bowel diseases and metabolic disorders [6,21]. Active targeting, utilising ligand–receptor interactions, enables preferential accumulation at disease sites [6].

### 5.6. Emerging Technologies

Emerging technologies include stimuli-responsive nanomaterials responding to pH, temperature, redox potential, enzyme activity, light, ultrasound, or magnetic fields [6]. Cell membrane-coated nanoparticles exhibit enhanced circulation stability and immune evasion [6]. Microfluidic manufacturing enables the production of monodisperse formulations with consistent batch-to-batch performance [6]. Successful clinical translation requires integration of formulation development with mechanistic pharmacology, regulatory compliance, and manufacturing scalability [6,21].

## 6. From Laboratory to Industry: Standardization and Scale-Up Challenges

Laboratory-to-industrial translation represents a critical bottleneck for phlorotannins. Despite extensive documentation of biological activities across pharmaceutical, nutraceutical, and cosmeceutical domains [1,2,22], industrial utilization remains limited. This disparity comes from unresolved technological hurdles: raw material inconsistency, heavy metal contamination, purification economics, delivery optimization, quality control standardization, and regulatory compliance [1,19,22]. Industries require consistent efficacy, shelf stability, reproducible quality, and stringent safety standards—factors currently overriding phlorotannins’ biological promise [1,22].

### 6.1. Raw Material Inconsistency and Standardization Crisis

The inherent ecological sensitivity of phlorotannin biosynthesis in brown macroalgae creates severe raw material variability, fundamentally complicating industrial standardisation and representing the primary upstream bottleneck in commercial development. Phlorotannin content in wild-harvested brown seaweed biomass fluctuates drastically from 0.5% to exceeding 30% of algae dry weight, depending on multiple interacting factors including species genotype, harvest location and depth, seasonal timing, water temperature, light intensity and spectral quality, salinity gradients, nutrient availability, herbivore grazing pressure, and physiological developmental stage [11,19].

**Figure 3:**
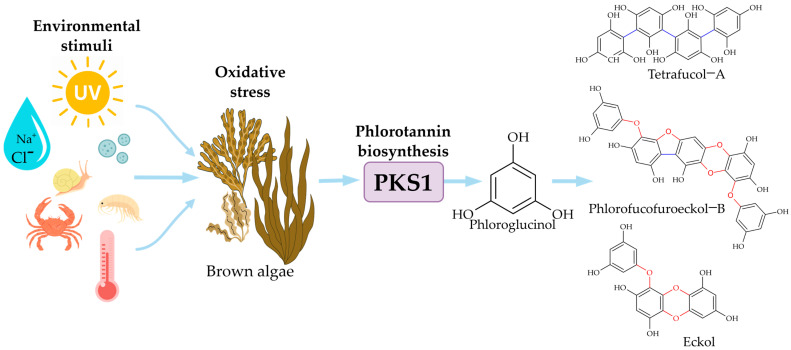
Conceptual diagram showing the influence of environmental stressors (UV radiation, salinity, microbial interactions) on the upregulation of phlorotannin biosynthesis in brown algae.

This extreme compositional variability directly translates into inconsistent extract potency, unpredictable bioactivity profiles, and unreliable therapeutic efficacy [22]. Phlorotannin biosynthesis is tightly regulated by environmental stressors that modulate oxidative stress responses and gene expression pathways in brown algae. Figure 3 conceptually illustrates the environmental stress-response cascade. Abiotic stressors including UV irradiation (280–320 nm), salinity fluctuations (10–40 PSU), temperature variations (5–25 °C), and biotic pressures (herbivory, microbial colonization) generate oxidative stress, triggering PKS1 gene upregulation and subsequent phlorotannin biosynthesis as a defensive adaptation [11,19,23].

Environmental stimuli including ultraviolet radiation, salinity fluctuations, temperature changes, and nutrient deficiency generate oxidative stress, triggering polyketide synthase type III (PKS1) gene activation [11,19]. This enzyme catalyzes phloroglucinol formation, with subsequent polymerization leading to structurally diverse phlorotannin classes (fucols, phlorethols, fucophlorethols, eckols) [11,19]. Abiotic stressors trigger adaptive upregulation of phlorotannin production, while biotic pressures such as herbivore grazing and epiphyte colonization necessitate differential metabolic resource allocation [11,12]. Seasonal variations in reproductive status further complicate phytochemical consistency [19].

This environmental sensitivity creates challenges for pharmaceutical and nutraceutical development requiring strict dosage consistency [22]. Quality control for wild-harvested sources necessitates comprehensive chemical fingerprinting using HPLC-MS for batch-to-batch assurance [7,19]. These requirements increase production complexity and manufacturing costs [19,22]. The feed industry has encountered similar standardization challenges when incorporating brown seaweed extracts as functional livestock additives, where phenolic variability has impaired reproducibility of antimicrobial effects [31].

### 6.2. Heavy Metal Contamination

Brown macroalgae accumulate heavy metals from seawater through bioconcentration mechanisms, presenting critical safety concerns [22]. Seaweeds from polluted coastal waters can contain elevated cadmium, lead, arsenic, and mercury concentrations posing nephrotoxicity, neurotoxicity, and carcinogenicity risks [22]. Research on *Durvillaea incurvata* extracts demonstrated necessity for 80% reduction in heavy metal content through upstream processing before biological evaluation [22]. Pre-processing requires specialized technologies including chelation protocols, activated carbon adsorption, ion-exchange chromatography, or membrane filtration [22]. Comprehensive analytical testing using ICP-MS or AAS verifies compliance with EFSA, FDA, and pharmacopeial limits [22]. Harvest location selection in pristine offshore waters away from industrial centers minimizes contamination risks [19].

### 6.3. Controlled Cultivation Environments

Controlled aquaculture cultivation represents the most promising solution to raw material inconsistency and contamination challenges [19,22]. Land-based recirculating aquaculture systems (RASs) and offshore farms using selective breeding enable phlorotannin standardization through precise control of water temperature, nutrient supplementation, light exposure, and salinity [19]. Strategic application of abiotic stress factors including controlled ultraviolet exposure and moderate nutrient limitation can enhance phlorotannin accumulation through predictable stress-response pathways [11,19]. Aquaculture site selection in pristine waters dramatically reduces heavy metal contamination compared to wild harvesting [19,22]. Selective breeding enables development of high-phlorotannin genotypes with enhanced biosynthetic capacity [19]. However, commercial-scale infrastructure requires substantial capital investment with return timelines extending over multiple years [19].

### 6.4. Purification Economics

Multi-step purification to isolate specific phlorotannin oligomers presents substantial scalability challenges [7,19]. Phlorotannin extracts contain heterogeneous mixtures of structurally related oligomers with varying polymerization degrees and overlapping physicochemical properties [4,7]. Industrial workflows require sequential separation techniques including liquid–liquid partitioning, Sephadex LH-20 size-exclusion chromatography, high-speed counter-current chromatography (HSCCC), and HPLC [7,33]. Each step introduces yield losses, increased processing time, and higher costs [7]. Extensive coelution of isomers sharing similar polarity and molecular weights remains a persistent challenge [7]. For pharmaceutical applications requiring high-purity compounds, cumulative costs can render products prohibitively expensive unless therapeutic efficacy justifies premium pricing [19]. Nutraceutical applications using partially purified extracts offer more favorable economics [19].

### 6.5. Industrial Applications and Market Status

**Figure 4:**
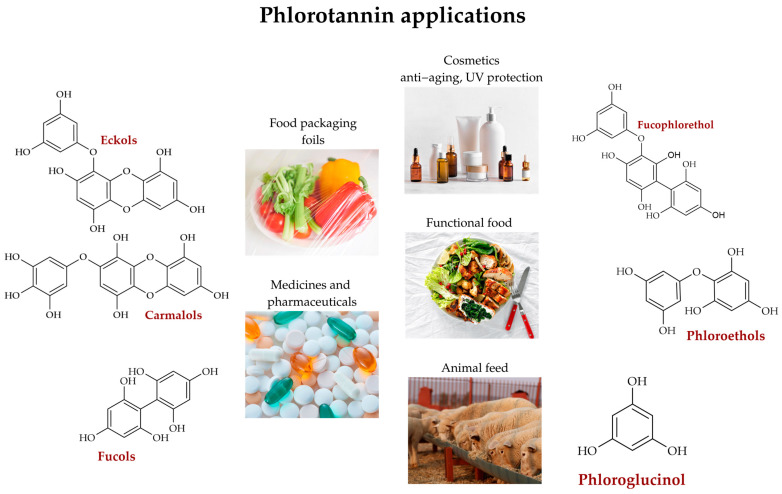
Overview of current and potential applications of phlorotannins in cosmetics, pharmaceuticals, functional foods, and biomaterials [4,10,37,50].

Phlorotannins exhibit exceptional potential for the development of high-value products across multiple industrial sectors (Figure 4), including pharmaceuticals, nutraceuticals, functional foods, cosmeceuticals, and veterinary medicine, due to their diverse biological activities, which encompass antioxidants, anti-inflammatory, antimicrobial, antidiabetic, anticancer, and neuroprotective mechanisms [1,2,6,47].

The global seaweed extracts market was valued at USD 2 billion in 2022, projected to reach USD 3.5 billion by 2032 at 6.1% CAGR, with phlorotannins driving pharmaceutical and nutraceutical growth [48]. The seaweed cosmetic ingredients market, valued at USD 500 million in 2023, is projected to reach USD 900 million by 2034 at 6.0% CAGR [49]. Phlorotannin-containing extracts from Fucus and *Ecklonia species* represent premium active ingredients [1,2,6,49].

Some of the phlorotannins are commercialized and their safety usage is regulated by appropriate agency approval (Table 2). The most commercially advanced phlorotannin product is SeaPolynol™ (marketed as Seanol^®^), a standardised *Ecklonia cava* extract containing ≥90% phlorotannins with defined eckol composition including dieckol, eckol, 6,6′-bieckol, 8,8′-bieckol, and phlorofucofuroeckol-A [51,52]. In 2008, the United States Food and Drug Administration (FDA) authorized SeaPolynol^®^ as a New Dietary Ingredient (NDI), permitting its marketing in dietary supplements at doses up to 47 mg phlorotannins/day for adults and children over 12 years [51]. In 2017, the European Food Safety Authority (EFSA) evaluated SeaPolynol™ as a Novel Food and concluded it is safe for use in food supplements at maximum daily intakes of 163 mg/day for adolescents aged 12–14 years, 230 mg/day for adolescents above 14 years, and 263 mg/day for adults, establishing a no-observed-adverse-effect-level (NOAEL) of 750 mg/kg body weight per day [52]. These regulatory approvals established *E. cava* phlorotannin extracts as the only legally tradeable phlorotannin ingredients in both US and EU dietary supplement markets [51].

Multiple commercial product formulations containing SeaPolynol^®^/Seanol^®^ have been developed and marketed internationally, including Seanol-F (standardized to 13–15% phlorotannins blended with dextrin for enhanced absorption), Seanol-P (high-purity formulation), Fibroboost, Circulate (cardiovascular support), Alginol, Memories with Seanol-P (cognitive support), Brilliant Vision with Seanol-P (eye health), Gly-Control (glycemic management), Lipid Balance (cholesterol support), and Marine D3 (vitamin D combination) [53]. These products are manufactured by Botamedi Inc. (South Korea) and distributed globally through nutraceutical channels. Commercial dietary supplements typically provide 50–400 mg of *Ecklonia cava* extract per serving, with retail prices ranging from $20–60 per 30–60 day supply depending on formulation complexity and phlorotannin standardization levels [51,53].

In the cosmeceutical sector, phlorotannin formulations provide anti-inflammatory, antioxidant, photoprotective, and anti-tyrosinase activities for skincare applications [1,2,54]. Brown seaweed extracts are listed in the European Commission’s Cosmetic Ingredients Database (CosIng) with 26 registered functions for pure seaweed and 42 functions for extract forms [55]. *Fucus vesiculosus* represents the most extensively used cosmetic species [55]. *Alaria esculenta* extract appears in commercial products from Glow Recipe, Korres, OSEA Malibu, Dermalogica, and L’Oréal [56]. Phlorotannin-enriched fractions from Brittany seaweeds (*A. esculenta*, *A. nodosum*, *F. serratus*, *Himanthalia elongata*) demonstrated antioxidant and photoprotective activities comparable to commercial molecules, with anti-aging activity exceeding epigallocatechin gallate [54]. The Cosmetic Ingredient Review Expert Panel concluded that brown algae-derived ingredients are safe for use in both leave-on and rinse-off products [45]. Major suppliers including BASF and Givaudan offer standardized brown seaweed extracts as active ingredients [50,55].

Nutraceutical applications target metabolic health, cardiovascular protection, and cognitive function [6,19]. As of 2023, over 1200 nutraceutical SKUs in Japan contained seaweed extracts [49]. Brown seaweed extracts are explored as functional additives in beverages, dairy products, and supplements [19]. Formulation challenges include stability during processing, organoleptic properties, and bioavailability [6,22]. Clinical evidence includes a randomized trial demonstrating *E. cava* extract (400 mg/day, 12 weeks) reduced total cholesterol by 2.8% and LDL-cholesterol by 11.1% in hypercholesterolemic patients [57]. A clinical trial showed dieckol-rich extract improved glycemic parameters in pre-diabetic individuals [58]. Phase IIa trial NCT04141241 evaluated PH100 at 800 mg/day and 1600 mg/day in patients with type 2 diabetes and cardiovascular complications [59]. Phase I studies established safety for doses from 100–1600 mg with no significant adverse effects [53].

The pharmaceutical sector requires extensive preclinical and clinical validation [6]. No phlorotannin-based formulations have achieved FDA or EMA approval [6]. Therapeutic targets include the inhibition of α-glucosidase and α-amylase, as well as the inhibition of acetylcholinesterase, ACE, and lipase [1,6,21]. Dieckol crosses the blood–brain barrier in preclinical models [60]. Clinical trials have not reported significant adverse effects [60]. Progression beyond Phase II remains limited by bioavailability challenges and manufacturing constraints.

Veterinary applications include the use of antimicrobial alternatives in livestock feed [31]. Phlorotannins demonstrate activity against *Escherichia coli* O157:H7 while reducing environmental zinc pollution [31]. In October 2024, BASF engaged with Acadian Plant Health for *Ascophyllum nodosum*-derived biostimulants [49].

Of approximately 150 identified phlorotannin structures, fewer than 5 appear in commercial products, predominantly as crude extracts [7]. Three factors explain this disparity: (1) purification costs ($50–200/g) exceed dietary supplement pricing ($5–15/g retail) [19]; (2) FDA/EMA require purified, characterized active ingredients unsuitable for crude extracts [6]; (3) no commercial aquaculture produces standardized high-phlorotannin biomass, with wild-harvested material exhibiting 500% batch-to-batch variability [11,19]. Market penetration remains limited despite substantial scientific foundation [1,2,19].

### 6.6. Quality Control and Analytical Standardization

Achieving consistent quality control in phlorotannin-containing products is a fundamental prerequisite for commercial success and regulatory approval, yet it remains challenging due to the inherent structural complexity, natural compositional variability, and limited availability of validated analytical methods and certified reference standards [7,31]. Establishing robust, reproducible protocols for extraction, purification, structural characterization, and quantitative analysis is essential for batch-to-batch consistency, accurate dosing, and meaningful cross-study comparisons [7,31].

Current analytical methodologies include spectrophotometric assays (Folin–Ciocalteu method), thin-layer chromatography (TLC), high-performance liquid chromatography (HPLC) with diode array or fluorescence detection, and HPLC coupled with electrospray ionization mass spectrometry (HPLC-ESI-MS) for structural identification [7]. However, validated studies dedicated to developing robust quantification methods, harmonized calibration protocols, and standardized quality control procedures suitable for regulatory submissions remain scarce [7].

A critical barrier is limited commercial availability of purified phlorotannin reference standards for method validation and calibration. Unlike well-established pharmaceutical compounds, most phlorotannin oligomers require custom isolation from algal biomass, creating supply constraints and cost barriers impeding standardized analytical protocol adoption [7].

High-resolution mass spectrometry with tandem fragmentation (MS/MS) provides enhanced sensitivity and specificity for characterizing complex phenolic structures [48]. However, integration into routine quality control workflows requires specialized instrumentation and expertise that may exceed smaller manufacturers’ resources [48].

International collaborative efforts toward analytical method harmonization, reference material repositories establishment, inter-laboratory validation studies, and consensus quality control guidelines development would significantly advance commercial standardization [7,31]. Implementation of comprehensive quality management systems incorporating Good Manufacturing Practices (GMP) and Hazard Analysis and Critical Control Points (HACCP) protocols represents essential infrastructure for commercial production [19,22].

## 7. Conclusions and Future Perspectives

This comprehensive review establishes that phlorotannins represent uniquely promising marine-derived bioactive compounds with demonstrated multi-target therapeutic potential across antioxidant, anti-inflammatory, antimicrobial, antidiabetic, anticancer, and neuroprotective mechanisms. However, clinical and commercial realization remains fundamentally constrained by three interconnected translational bottlenecks rather than insufficient bioactivity documentation. The bioavailability paradox manifests as acute discrepancy between potent in vitro activities and negligible oral bioavailability due to physicochemical instability, limited aqueous solubility, gastrointestinal degradation, and rapid metabolic clearance. The standardization crisis reflects extreme ecological sensitivity of phlorotannin biosynthesis, generating severe raw material variability that fundamentally complicates quality control and regulatory approval. The delivery system bottleneck persists because advanced formulation technologies remain at proof-of-concept stages, with no phlorotannin nanodelivery systems progressing beyond Phase I trials.

Five critical research priorities emerge as essential for achieving industrial scalability, formulation reproducibility, and regulatory acceptance. Standardized production through controlled aquaculture represents the highest-impact priority, requiring establishment of commercial-scale cultivation systems with environmental parameter control, selective breeding for high-phlorotannin genotypes, real-time metabolomic monitoring, and pristine offshore site selection to reduce batch-to-batch variability from current 500% to under 20%. Advanced delivery system clinical validation constitutes the most urgent priority, demanding systematic comparative bioavailability studies evaluating PEGylated liposomes, PLGA nanoparticles, and pH-responsive enteric systems using purified standards, comprehensive preclinical ADME characterization, and Phase I safety trials for lead platforms to achieve 5–10-fold bioavailability enhancement. Mechanistic pharmacology requires systematic structure–activity relationship studies correlating specific structural features with molecular target affinity, detailed pathway elucidation through integrated omics approaches, identification of critical circulating metabolites through human pharmacokinetic studies, and predictive computational modelling enabling rational analogue design.

Harmonised analytical standards demand international reference material repositories, consensus method validation through multi-laboratory trials achieving under 15% inter-laboratory variation, industry-wide GMP guidelines aligned with FDA botanical drug guidance, and advanced orthogonal analytical approaches combining UHPLC-MS/MS with quantitative NMR. Long-term safety characterisation necessitates complete regulatory toxicology batteries including 90-day chronic studies, reproductive and developmental toxicity assessments, comprehensive genotoxicity panels, drug interaction investigations, and post-market surveillance generating real-world safety data.

Successful clinical translation requires coordinated, parallel advancement across all five priorities through integrated translational research consortia bringing together academic researchers, aquaculture industry, pharmaceutical formulation specialists, analytical laboratories, and regulatory consultants. Prioritised funding should emphasise delivery system development and standardised production as rate-limiting bottlenecks with the highest immediate impact while simultaneously building long-term foundations through mechanistic research, analytical harmonisation, and safety characterisation. The marine bioactive sector’s historical pattern of extensive basic research yielding minimal commercial translation must be disrupted through deliberate strategic focus on translational enablers rather than continued bioactivity discovery.

Phlorotannins possess exceptional therapeutic potential, but realizing this potential demands systematic resolution of fundamental pharmaceutical development challenges through sustained, coordinated investment in the translational research priorities outlined above.

## Figures and Tables

**Table 1:** Structural classification of phlorotannins based on linkage type.

Phlorotannin Type	Algal Source	Extraction Method	Biological Activities/Applications	Remarks	References
Fucols	*Fucus vesiculosus*	Aqueous ethanol, solid–liquid	Prebiotic effect, UV-radiation protection	C–C linked phloroglucinol units; abundant in temperate brown algae	[2]
Phlorethols	*Ascophyllum nodosum*	Ethanol–water + ultrasound	Antioxidant, neuroprotective	Ether linkages; higher solubility than fucols	[3]
Fucophlorethol	*Sargassum muticum*	Deep eutectic solvent (DES) + ultrasound	Antioxidant, enzyme inhibition	Complex mixture; mixed linkages	[36]
Eckol	*Ecklonia cava* (Lessoniaceae/Phaeophyta)	Methanol extraction; SPE purification	Antidiabetic, UV-protection, neuroprotective	Dibenzo-1,4-dioxin ring	[1]
Dieckol	*Ecklonia cava*/*E. stolonifera*	Enzyme-assisted extraction + SPE	Anti-obesity, anti-photoaging, tyrosinase inhibition	Hexamer phlorotannin	[37]
Carmalol	*Carpophyllum maschalocarpum*	Ethanol extraction	Unstable under gastrointestinal digestion; antioxidant potential	Dibenzodioxin-linkage subclass of phlorotannins	[38]
Miscellaneous polymeric phlorotannins	Various Fucaceae, Sargassaceae, Alariaceae	Pressurized liquid extraction (PLE)/MAE/DES	Broad-spectrum antioxidant, UV-shielding	Very high degree of polymerization; structural diversity	[18]
Undaria-specific phlorotannins	*Undaria pinnatifida* (Alariaceae)	Microwave-assisted extraction (MAE)	Skin-whitening, anti-inflammatory (cosmeceutical)	Commercial interest in Asia	[39]

**Table 2:** Commercial phlorotannin products, algal sources, and regulatory status.

Product Name	Algal Source	Key Compounds	Standardization	Regulatory Status	Applications
SeaPolynol™/Seanol^®^	*Ecklonia cava*	dieckol, eckol, 6,6′-bieckol, PFF-A	≥90% total phlorotannins	FDA NDI (2008); EFSA Novel Food (2017)	Dietary supplements, cardiovascular, cognitive
Seanol-F	*E. cava*	eckol derivatives	13–15% phlorotannins with dextrin	FDA NDI (2008)	Enhanced absorption supplements
Ventol^®^	*E. cava*	phlorotannin-rich extract	proprietary	Korea FDA approved	Herbal medicine
Pepha-Tight^®^	*Nannochloropsis oculata* (microalgae)	polysaccharides, phenolics	film-forming matrix	CIR-approved cosmetic	Anti-aging skincare
Fucus/Ascophyllum extracts	*F. vesiculosus*, *A. nodosum*	fucols, phlorethols	variable (1–15% phenolics)	CosIng listed; GRAS	Cosmetics, agriculture

FDA—Food and Drug Administration; NDI—New Dietary Ingredient; EFSA—European Food Safety Authority; PFF-A—phlorofucofuroeckol-A; CIR—Cosmetic Ingredient Review; GRAS—Generally Recognized As Safe; CosIng—Cosmetic Ingredient Database.
